# Significance of HLA in Graves’ disease and Graves’ orbitopathy in Asian and Caucasian populations – a systematic review

**DOI:** 10.3389/fimmu.2023.1256922

**Published:** 2023-09-28

**Authors:** Magdalena Stasiak, Bartłomiej Stasiak, Katarzyna Zawadzka-Starczewska, Andrzej Lewiński

**Affiliations:** ^1^ Department of Endocrinology and Metabolic Diseases, Polish Mother’s Memorial Hospital—Research Institute, Lodz, Poland; ^2^ Institute of Information Technology, Lodz University of Technology, Lodz, Poland; ^3^ Department of Endocrinology and Metabolic Diseases, Medical University of Lodz, Lodz, Poland

**Keywords:** Graves’ disease, Graves’ orbitopathy, human leukocyte antigen, HLA, Asian population, Caucasian population, genotyping

## Abstract

**Introduction:**

Graves’ disease (GD) and Graves’ orbitopathy (GO) development were suspected to be HLA-related in both Asian and Caucasian populations. However, most studies were performed with application of serological methods or low resolution genetic typing, which led to inconsistent results even among the same population. The present review is intended to summarize the state-of-art knowledge on the HLA significance in GD and GO in Asians and Caucasians, as well as to find the most significant alleles for each of the populations.

**Methods:**

PubMed was searched for relevant articles using the following search terms: HLA plus thyroid-associated ophthalmopathy or Graves’ disease or Graves’ orbitopathy or thyroid eye disease or thyroid-associated orbitopathy.

**Results:**

In Asian population GD was found to be associated mostly with B*46:01, DPB1*05:01, DRB1*08:02/03, DRB1*16:02, DRB1*14:03, DRB1*04:05, DQB1*05:02 and DQB1*03:03, while DRB1*07:01, DRB1*01:01, DRB1*13:02, DRB1*12:02 are potentially protective. HLA-B*38:02, DRB1*16:02, DQA1*01:02, DQB1*05:02 can be considered associated with increased risk of GO in Asians, while HLA-B*54:01 may play protective role. In Caucasians, C*07:01, DQA1*05:01, DRB1*03, DQB1*02:01 are associated with GD risk while DRB1*07:01, DQA1*02:01 may be protective. Significance of HLA in the course of GD and novel aspects of HLA amino acid variants and potential HLA-based treatment modalities were also discussed.

## 1 Introduction

Graves’ disease (GD) is an autoimmune thyroid disorder caused by production of antibodies against thyrotropin (TSH) receptor. TSH-receptor antibodies (TRAb) usually stimulate thyroid hormone production, but they can also block TSH-receptor (TSHR) or have ambivalent character with no impact on thyroid function (1). Additionally, insulin-like growth factor-1 (IGF-1) receptor (IGF-1R) was demonstrated to play an important role in GD development (2). An activation of IGF-1R on orbital fibroblasts may result from the binding of stimulatory IGF-1R antibodies (IGF-1R-Ab) to IGF-1R. Synergistic crosstalk between TSHR and IGF-1R after binding of stimulatory TRAb to TSHR may also be a mechanism leading to IGF-1R activation (3). The prevalence of GD in general population is about 0.5-2.0% (1, 4). Like many other autoimmune diseases, GD is typically induced by environmental factors in genetically predisposed individuals (5, 6). Graves’ orbitopathy (GO) is the most frequent extrathyroidal manifestation of GD, with the estimated incidence of 2.67–3.3 cases/100 000/year in women and 0.54–0.9 cases/100 000/year in men (7). GO significantly deteriorates patients’ quality of life (QoL) and may even lead to a sight-threatening conditions (7).

Therefore, the knowledge on risk factors associated with GD and GO development seems to be crucial for the prevention and management of GO. Many environmental agents were demonstrated to be potential GD-triggering factors, including viral infections, vaccines (8), drugs (9), stress (5), reactive oxygen species (ROS) overproduction (10). Smoking, high serum TRAb levels, severe or unstable hyperthyroidism as well as hypercholesterolemia are already known risk factors of GO development and progression (7, 11).

However, as not all exposed individuals are affected and – on the other hand – GD and GO are more frequent among family members, it seems obvious that environmental factors always act on a genetic susceptibility which is crucial for the disease development. The strength of genetic susceptibility was proven in environmental, family and twin studies, which suggested that 70% of the risk of GD can be attributed to genetic factors (12). Among genes associated with the autoimmune response, human leukocyte antigen (*HLA*) genes seem to play a prominent role as a molecular background of GD and GO (13–15). Many different HLA alleles were postulated as GD and/or GO risk factors in Caucasian and Asian populations. The role of other genes such as *TSHR*, cytotoxic T lymphocyte-associated factor 4 (*CTLA-4*), protein tyrosine phosphatase non-receptor type 22 (*PTPN22*), Fc receptor like 3 (*FCEL3*), interleukin 2 receptor A (*IL2RA*), thyroglobulin (*TG*) or cluster of differentiation 40 (*CD40*) genes was also postulated in GD development (16–19). Similarly, the risk of GO occurrence in GD patients is associated with genetic susceptibility. Except for HLA, several other genes were suggested as GO-related, including *TSHR*, *CTLA4*, tumor necrosis factor (*TNF*), interferon γ (*IFN γ*), interleukin 1A (*IL1A*) and its receptor (*IL1AR*), protein tyrosine phosphatase non-receptor type 12 (*PTPN12*), peroxisome proliferator-activated receptor γ (*PPAR γ*) or intracellular adhesion molecule 1 (*ICAM-1*) genes (4, 20–24). There is evidence that the risk of GD and/or GO is not related to a single gene, but is a result of a complex interaction between genetic factors (4). Nevertheless, considering the importance of the major histocompatibility complex (MHC) for autoimmune responses and taking into account high polymorphism of HLA region, HLA seems to play a prominent role as a molecular background of GD and GO (4, 14, 15).

However, results of studies regarding either Caucasian or Asian population were not coherent. The major reasons of this inconsistency are: different sizes of study groups and different methods used by the researchers, including low resolution or serological methods (13). Serological methods detect antigens only, so their accuracy is very low and their application is currently not recommended. More precise results can be obtained with DNA typing methods for HLA analysis. These include i.a.: polymerase chain reaction restriction fragment length polymorphism (PCR-RFLP), PCR sequence-specific oligonucleotide probe (PCR-SSOP), PCR sequence-specific primer (PCR-SSP), PCR single-strand conformation polymorphism (PCR-SSCP), and sequence-based typing (SBT). These methods, except for the SBT, require more probes and primers to maintain the acceptable (low or intermediate) resolution, and are unable to detect new polymorphism (25). On the other hand, SBT method can identify all sequence motifs and is capable of detecting new undefined alleles (25). Low resolution methods provide results for the entire allelic group, but not for a particular allele. Serological methods or low resolution DNA-based methods of HLA typing provide results only at antigen or allelic group level. The quality of the results was significantly improved by high-resolution DNA-based typing. In order to further improve this quality, next-generation sequencing (NGS) methods which are based on deep-sequencing of the entire HLA gene followed by bioinformatic processing, have been introduced to provide typing results at the allelic level (25, 26). The use of serological or low resolution methods may lead to erroneous conclusions and inconsistency in the study results depending on the applied method. More and more studies which demonstrated method-dependent error in HLA analysis are available. Among a strictly controlled cohort in whom HLA typing was performed for bone marrow transplantation, discrepancies exceeding 29% were reported between less sensitive methods and NGS method (27). Another important example of the risk related to low resolution methods is HLA-B*27 test, which is commonly used to confirm a diagnosis of ankylosing spondylitis. However, it has been recently demonstrated that alleles HLA-B*27:06 and HLA-B*27:09 are probably not associated with the disease, so the results based on less precise methods may lead to a wrong diagnosis (28). Therefore, the significance of applied HLA-typing method seems crucial to unequivocally determine HLA-related background of autoimmune diseases, including GD.

The purpose of this review is to summarize the current knowledge on the HLA significance in GD and GO in Asians and Caucasians, as well as to indicate the most reliable sets of high risk-related or protective alleles for each of the populations. Identification of an actual sets of such alleles can constitute a reliable tool for the individual risk assessment, and would play an important role in a development of personalized medicine. Additionally, other aspects of HLA significance in GD/GO were also summarized.

## 2 Methods

PubMed was searched for relevant articles using the following search terms: HLA plus one of the following: thyroid-associated ophthalmopathy or Graves’ disease or Graves’ orbitopathy or thyroid eye disease or thyroid-associated orbitopathy. Studies which analyzed HLA-associated susceptibility to GD were included only if comparison with healthy control group was performed. Results based exclusively on comparison with patients with other autoimmune diseases, without a control group, were excluded. In the case of HLA-related background of GO, only studies which provided comparison between GD patients with GO and without GO were included. A total number of 1197 studies were found through initial database searching. Subsequently, after thorough screening of titles and abstracts, 368 studies were selected after exclusion of duplicates, articles not available in English, or in full text, and irrelevant papers. These studies were reviewed in detail. Two hundred seventy two of these were excluded as they did not meet the inclusion criteria or due to data overlapping, insufficient data, or for other reasons. Study selection flowchart is available as a Supplementary Material. A total number of 96 papers were included and, on the basis of the studied population and analyzed factors, these papers were divided into the following groups of papers 1. Results on HLA-related GD risk in Asians, 2. Results on HLA-related GO risk in Asians, 3. Results on HLA-related GD risk in Caucasians, 4. Results on HLA-related GO risk in Caucasians, 5. Results on other correlations between HLA and GD/GO (recurrence risk, clinical course, amino acid variants significance etc.). For further analysis of Group 1 and Group 3, only studies performed with genetic (not serological) methods were included, and only alleles which were reported as related to increased or decreased risk in more than one study were presented as summary results.

## 3 Results

### 3.1 Population-dependent differences and comparison difficulties

An importance of significant ethnic differences was indicated in many studies on HLA-related susceptibility to GD (5, 6, 29–34). Furthermore, the results already published for either Asian or Caucasian populations are inconsistent (6, 29–34). Additionally, the symbols of alleles used for the previous methods differ from the ones currently used. Application of high-resolution methods changed the obtained results significantly, because antigens previously denoted by a given symbol have been separated into many different alleles (13). This fact obviously highly influenced the accuracy and coherence of the already published results.

### 3.2 Asian population

#### 3.2.1 HLA and GD development

Many studies on the potential significance of HLA for GD in Asians were performed. Since 1978, several of them have postulated the impact of HLA-B*46 in the development of GD (31–33). However, with application of serological methods, in some studies, HLA*Bw46 significance was demonstrated in males only, with lack of correlation in females (34–36). Similar gender-limited relationship was observed between GD and HLA-DR9 and DQB1*03:03 (36). A metanalysis of 14 studies performed by Li et al. in 2013 demonstrated association between HLA-B*46 and GD in Asians (31). Although, most of the authors of the included studies applied serological methods, more recent reports confirmed this correlation (37–40). The overview of the published results on HLA significance in GD are demonstrated in **Table 1**. It is clearly visible that the obtained results are not consistent and potential high risk alleles or protective ones are different even in similar populations (i.e. in the Chinese, the Japanese, the Korean etc.). Several antigens other than Bw46 were postulated by authors who used serological methods, including, for example, HLA-DRw8, -DQw4, -B5, Dw12 and –A11 antigens (44–46). At the end of the 20th century, polymerase chain reaction (PCR) sequence specific oligonucleotide probe (SSOP) method was introduced and many authors applied it at least for MHC class II analysis. Similarly to the serology-based reports, the studies with combined serological and SSOP methods revealed inconsistent results. Chan et al. reported that the risk of GD was higher in patients with HLA-A*2, Cw1, DRB1*16:02, DRB1*03:01, DRB1*14:05, DRB5*02, DQB1*05:02, while the presence of HLA-DRB1*15:01 and -DQB1*03:01 played a protective role (35). On the other hand, Japanese authors presented that the most important factor was the presence of HLA-DPB1*05:01 and/or HLA-A*2, with the risk being the highest in carriers of both of them (46). In one Taiwanese study, HLA-A*02:07 was found a GD risk factor (37), while other Taiwanese authors showed correlation between GD and HLA-B*46:01, DPB1*05:01, DQB1*03:02, DRB1*15:01 and DRB1*16:02, with the strongest relationship existing with HLA-DPB1*05:01 (38).

**Table 1:** Associations between HLA and Graves’ disease in Asian population.

First author	Ref.	Year	Population	Method	No of GD patients	No of controls	Risk antigens/ Alleles	Protective antigens/allels
Chan SH	(41)	1978	Chinese	serological	86	238	Bw46	NR
Hawkins BR	(32)	1985	Chinese	serological	132	110	Bw46 (younger), B5 (older)	NR
Naito S	(33)	1987	Japanese	serological	61	1998	Bw46 CX46 (Cw1+Cw3)	A24, Cw3
Cho BY	(42)	1987	Korean	serological	128	220	B13 DR5 DRw8	NR
Tamai H	(43)	1987	Japanese	serological	35	263	HLA-A2, Cw3, DRw8	NR
Yeo PP	(34)	1989	Chinese	serological	159	330	Bw46 (significance for males), DRw9 (significance for males)	NR
Tsai KS	(44)	1989	Chinese	serological	93	106	DR2 DR9 DRw53 DQw1	DR3 Rw52
Inoue D	(45)	1992	Japanese	HLA-A, -B, -C, -DR and -DQ loci – serological HLA-D, -DP by RFLP	88	186	Bw46, Bw48, DRw8, DQw3, DQw4	A31, DRwl3, DPw2
Dong RP	(46)	1992	Japanese	HLA-A, B, C, DR, and DQ by serologic typing HLA-DPB1 by SSOP	76	317	A2, B46,Cw11, DPB1*05:01	HLA-A24, B7, Bw52, and DR1
Chan SH	(35)	1993	Chinese	serological and SSOP	33	79	B46 (significance for males only) A2, Cw1 DRB1*16:02 DRB1*03:01 DRB1*14:05 DRB5*02 DQB1*05:02	A24, B63 DRB1*15:01 DQB1*03:01
Onuma H	(47)	1994	Japanese	PCR-RFLP	106	100	B46 (significance for late onset only) DPB1*05:01(significance for early onset only)	NR
Cavan DA	(36)	1994	Chinese	Serological for HLA-A,B, DR, SSOP for DQA1, DQB1	97	105	HLA-B46 (significance for males only) DR9 (significance for males only) DQB1*03:03 (significance for males only)	DR12, DQA1*04:01
Ohtsuka K	(48)	1998	Japanese	SSOP	94	767	DQB1*03:03 (only children included)	DQB1*02:01 (only children included)
Wong GW	(49)	1999	Chinese	SSOP	67	51	DQB1*03:03 (only children included)	DQB1*02:01 (only children included)
Huang SM	(37)	2003	Chinese	SSOP	236	533	A*02:07 B*27:04, B*46:01 DRB1*09:01	Haplotype: A*33:03-B*58:01-DRB1*03:01
Park MH	(50)	2005	Korean	SSOP	198	200	DQB1*05:02 DQB1*06:01 DRB1*08:03 DRB1*16:02	DRB1*01:01 DRB1*13:02 DRB1*12:02 DRB1*07:01 DQB1*02:02 DQB1*05:01 DQB1*06:04
Iwama S	(51)	2005	Japanese	SSOP	43	608	DRB1*04:05 DQB1*04:01	NR
Wongsurawat T	(52)	2006	Thai	SSP for DQA1 and DQB1; SSOP for HLA-DRB1	124	124	DRB1*16:02 DQA1*01:02 DQB1*05:02	DRB1*07 DQA1*02:01 DQA1*06:01
Takahashi M	(53)	2006	Japanese	PCR-SSP, HLA-DPB1 by PCR-RFLP	48	321	A*02:06, DPB1*05:01	NR
Cho WK	(54)	2011	Korean	PCR-SSP	41	159	HLA-A*02 B*46 Cw*01 DRB1*08	DRB1*07 Cw*07
Chen PL	(55)	2011	Chinese	PCR-SSOP	499	504	B*46:01, DPB1*05:01 DQB1*03:02 DRB1*15:01 DRB1*16:02	DRB1*12:02
Jang HW	(56)	2011	Korean	PCR-SBT	133	200	DRB1*03:01 DRB1*08:02 DRB1*14:03	DRB1*07:01 DRB1*13:02
Ueda S	(39)	2014	Japanese	NGS	547	481	B*35:01, B*46:01, DRB1*14:03 DPB1*05:01	A*24:02, A*33:03, C*12:02, C*14:03, B*07:02, B*44:03, B*52:01 DRB1*01:01 DRB1*13:02 DRB1*15:02 DQB1*05:01 DQB1*06:04 DPB1*04:01 DPB1*09:01
Shin DH	(14)	2019	Japanese	NGS	106	142	HLA-B*46:01 HLA-C*01:02 DPB1*02:02 DPB1*05:01	*-*
Katahira M	(57)	2021	Japanese	NGS	243	82 HT	DRB1*04:05 DRB1*14:03	DRB1*01:01, DRB1*15:02
Liao WL	(40)	2022	Chinese	NGS	2047	29083	*Genotypes:* A*11:01-*11:01 A*02:07-*11:01 B *40:01-*46:01 B*46:01-*46:01 C *01:02-*01:02 C*01:02-*03:04 C*01:02-*07:02 *DPA1* *02:02-*02:02 *DPB1* *02:01-*05:01 *02:02-*05:01 *04:01-*05:01 *DQA1* *01:02-*03:02 *03:02-*05:05 *DRB1* *04:05-*09:01 *09:01-*11:01 *09:01-*15:01 *09:01-*09:01	*Genotypes* A*11:01-*33:03 A*24:02-*33:03 A*24:02-*24:02 B*40:01-*58:01 C*03:02-*03:04 *DPA1* *01:03-*01:03 *02:01-*02:02 *01:03-*02:01 *DQA1* *03:02-*06:01 *01:02-*06:01 *DQB1* *03:01-*03:01 *DRB1* *09:01-*12:02

NR, not reported, PCR, polymerase chain reaction; RFLP, restriction fragment length polymorphism; SBT, sequence-based typing; SSOP, sequence specific oligonucleotide probe; SSP, sequence specific primers; NGS, next generation sequencing.

There are discrepancies in the obtained results, not only between methods applied by the authors or between subpopulations based on patients’ nationality, but also between studies performed with the same method and among the same subpopulation (40–57). Additionally, many studies included MHC class II alleles only, or even one group mostly HLA-DRB1 (48–52). Therefore, direct comparison of the results, or any attempt to include the studies into metanalysis, would be subjected to high risk of error. The most reliable results obtained with NGS method are scarce. Katahira et al. did not compare their GD patients to a control group but only to patients with Hashimoto thyroiditis. Therefore, they are not suitable for comparison with others. Ueda et al. presented results as a list of alleles associated with high risk of GD and a list of 3 haplotypes which showed significant protective effects against the development of GD in Japanese population (HLA-A*24:02-C*12:02-B*52:01-DRB1*15:02-DQB1*06:01-DPB1*09:01 and HLA-A*24:02-C*07:02-B*07:02-DRB1*01:01-DQB1*05:01-DPB1*04:02, and HLA-A*33:03-C*14:03-B*44:03-DRB1*13:02-DQB1*06:04-DPB1*04:01). Taking into account the difficulties in direct comparison of the results, we decided to analyze a number of studies which revealed a given allele as the high risk one or the protective one. In order to obtain the most reliable results we included all studies which used methods which allowed to obtain allelic specificity. We did not include any of the results obtained by serological methods. As it was stated above, a metanalysis of those studies previously confirmed a significance of HLA-B46 only.

Our current analysis demonstrated that the following alleles were most commonly reported as related to high risk of GD in Asians: B*46:01 (7 studies), DPB1*05:01 (7 studies), DRB1*16:02 (4 studies), DRB1*08:02/03 (4 studies), DRB1*14:03 (3 studies), DRB1*04:05 (3 studies), DQB1*05:02 (3 studies), DQB1*03:03 (3 studies, including two studies with significance for children only and one study with significance for males only) (**Table 2**). On the other hand, the following alleles were reported as potentially protective: DRB1*07/DRB1*07:01 (4 studies), DRB1*01:01 (3 studies), DRB1*13:02 (3 studies), DRB1*12:02 (3 studies) (**Table 2**).

**Table 2:** Alleles reported as Graves’ disease (GD)-related in more than one study in Asian population.

GD risk alleles	No. of papers	Ref.	GD protective alleles	No. of papers	Ref.
B*46/B*46:01:01	7	(14, 37, 39, 40, 47, 54, 55)	DRB1*07/DRB1*07:01	4	(50, 52, 54, 56)
DPB1*05:01	7	(14, 39, 40, 46, 47, 53, 55)	DRB1*01:01	3	(39, 50, 57)
DRB1*16:02	4	(35, 50, 52, 55)	DRB1*13:02	3	(39, 50, 56)
DRB1*08/ DRB1*08:02/03	3	(50, 54, 56),	DRB1*12:02	3	(40, 50, 55),
DRB1*14:03	3	(39, 56, 57)	A*24:02	2	(39, 40)
DRB1*04:05	3	(40, 51, 57)	A*33:03	2	(39, 40)
DQB1*03:03	3	(36) (males only) (48, 49) (children only)	B*58:01	2	(37, 40)
DQB1*05:02	3	(35, 50, 52)	DRB1*15:02	2	(39, 57)
A*02:07	2	(37, 40)	DQB1*02:01 (children only)	2	(48, 49) (children only)
C*01:02	2	(14, 40)	DQB1*05:01	2	(39, 50)
DRB1*03:01	2	(35, 56)	DQA1*06:01	2	(40, 52)
DRB1*15:01	2	(40, 55)	DQB1*06:04	2	(39, 50)
DRB1*09:01	2	(37, 40)	DQB1*03:01		(35, 40)
DQA1*01:02	2	(40, 52)			
DPB1*02:02	2	(14, 40)			

It is worth indicating, that among the alleles found as related to high risk of GD on the basis of our present review, HLA-DRB1*16:02 is in linkage disequilibrium with DQB1*05:02 (58), so they cannot be considered independent risk predictors. Additionally, among alleles demonstrated as related to lower risk *HLA*-DRB1*01:01 is in linkage disequilibrium with DQB1*05:01, DRB1*12:02 is in linkage disequilibrium with DQA1*06:01 and DRB1*13:02 is in linkage disequilibrium with DQB1*06:04 (58). These correlations must be taken into account if independent associations are analyzed.

#### 3.2.2 HLA and GO development

Similarly to GD, the results concerning HLA associations with GO development are inconsistent. The overview of the published results is presented in **Table 3**. Inoue et al. used serological method in Japanese patients and demonstrated that only DQw3 seemed to be a GO risk factor (59). However, in a study published a year later, Inoue et al. found that the risk of GO was associated with the following sets of HLA antigens: HLA-DQw4 without presence of -A31, HLA-A11 without presence of -DPw2, and with a co-presence of HLA-B5 and -Dw12 (45). Other authors applied serological methods, in another group of patients from Japan, and obtained entirely different results (60). They reported that predisposition to severe GO can be related to a presence of HLA-DR14 and DQ1 antigens, while HLA-B35, B54, DR4, and DQ4 were postulated to play a protective role (60).

**Table 3:** Associations between HLA and Graves’ orbitopathy in Asian population.

First author	Ref.	Year	Population	Method	No. of GO patients	No. of GD patients	No. of healthy controls	High risk HLA	Protective HLA
Inoue D	(59)	1991	Japanese	serological	23	88	186	DQw3, DPw2	NR
Inoue D	(45)	1992	Japanese	serological	42	88	186	-DQw4 (+) and -A31 (–) -B5 (+) and -Dw12 (+) -A11 (+) and -DPw2 (+)	NR
Ohtsuka K and Nakamura Y	(60)	1998	Japanese	serological	48	94	767	DR14, DQ1	B35, B54, DR4, DQ4
Jang HW	(56)	2011	Korean	PCR-SBT	13	120	200	DRB1*12:01	NR
Mehraji Z	(61)	2017	Iranian	SSP	45	80	180	none	NR
Shin et al.	(14)	2019	Korean	NGS	35	71	142	C*03:03	B*54:01
Huang et al.	(62)	2021	Chinese	NGS	82	272	411	B*38:02 DRB1*16:02 DQA1*01:02 DQB1*05:02	NR

PCR, polymerase chain reaction; SBT, sequence based typing; SSP, sequence specific primers; NGS, next generation sequencing; NR, not reported.

In Iranian cohort, no differences in allele distribution between patients with and without GO was postulated (61).On the contrary, in a Korean study, HLA-C*03:03 allele had higher frequency of occurrence in GO as compared to non-GO patients (14). In a Chinese cohort described by Huang et al., the HLA-B*38:02, DRB1*16:02, DQA1*01:02 and DQB1*05:02 were postulated as GO high risk alleles (62). No consistent results were found (**Table 3**), therefore, no allele can be unequivocally selected as actually GO related. Taking into account the significance of the applied method and the size of groups, the results presented by Huang et al. in 2021 can be considered the most reliable, as they were performed by NGS method in 82 GO patients, 272 GD patients and 411 healthy controls (**Table 3**) (62). Therefore the alleles HLA-B*38:02, DRB1*16:02, DQA1*01:02 and DQB1*05:02 can be considered associated with increased risk of GO in Asians, although such relationship was demonstrated in one study only and requires further confirmation. Moreover, HLA-DRB1*16:02 is in linkage disequilibrium with DQA1*01:02 and DQB1*05:02 (58), so their associations with GO cannot be considered independent. On the other hand, the significance of HLA-B*38:02 seems to be entirely independent. In this study, no potentially protective alleles were selected, however there was a concordance in two previous studies in regard to HLA-B*54/HLA-B*54:01 which was demonstrated to play a protective role (14, 60). Therefore HLA-B*54:01 can be considered as associated with lower GO risk in Asian population.

### 3.3 Caucasian population

#### 3.3.1 HLA and GD development

In Caucasians, GD was initially reported to be associated with allelic group DRB1*03, i.e. with B8 and Dw3antigens (63). Many serological studies performed between 1978 and 1991 confirmed the significance of B8and DRw3 (63–75). In different Caucasian populations, the frequency of DRB1*03 in GD patients was demonstrated to range between 40% and 55%, while in the general population it ranged between 15% and 30%. The relative risk (RR) of GD in DRB1*03 carriers was assessed as approximately 4 (76). Since 1993, HLA-DQA1*05 has been reported as associated with GD (77, 78) and a few years later, HLA-DQB1*02 was also demonstrated to be related to GD in Caucasians (6). For many years, the results of studies were concordant mainly with regard to the increased risk of GD in carriers of HLA-DRB1*03 and of the alleles DQA1*05:01, DQB1*02:01 which are in linkage disequilibrium with HLA-DRB1*03 in Caucasians (6, 74, 77). Both case-control and family studies, demonstrated that a haplotype DRB1*03- DQA1*05-DQB1*02 can be considered a predictor of the development of GD (78).

However, HLA-DRB1*03 is well known to be associated with an increased risk of many autoimmune diseases, not exclusively with GD. Increased risks of Hashimoto’s thyroiditis, myasthenia gravis, Addison’s disease, diabetes mellitus type 1, systemic lupus erythematosus (SLE) were reported (79).

Many other alleles were postulated, but hardly any consistency was found between the results. Vita et al. found that frequencies of HLA-C*07, -C*17 and -DRB1*04 are significantly higher in patients with GD than in controls (5), while Heward et al. suggested that HLA-DQB1*03:01/04 and -DQB1*02 can play a role in GD occurrence (6).

Recently, the significance of B*08:01, B*39:06, B*37:01, C*07:01, C*14:02, C*03:02, C*17:01, DRB1*03:01, DRB1*11:01, DRB1*13:03, DRB1*01:03, DRB1*14:01, DQB1*03:01, DQB1*02:01 was demonstrated by our research team with application of NGS method (13). Unfortunately, it was the first study which applied NGS in Caucasian patients, therefore, no comparison with other NGS-based studies is possible. The overview of studies focused on associations between HLA and GD in Caucasian population is presented in **Table 4**.

**Table 4:** Associations between HLA and Graves’ disease in Caucasian population.

First author	Ref.	Year	Population	Method	No of GD patients	No of controls	Risk antigens/ Alleles	Protective antigens/ alleles
Bech K	(63)	1977	Danish	serological	86	1967	B8 Dw3 (both in patients with GD relapse only)	NR
Farid NR	(64)	1979	Canadian	serological	41	50	DRw3 B8 DRw2	NR
McGregor A	(65)	1980	British	serological	65	325	DRw3	NR
Farid NR	(66)	1980	Canadian	serological	175	222	DRw3 B8	NR
Allannic H	(67)	1980	French	serological	86	100	DRw3 B8 A1	B12
Dahlberg PA	(68)	1981	Swedish	serological	78	100	DR3 B8	NR
McKenna R	(69)	1982	Irish	serological	86	95	DR3 B8	NR
Allanic H	(70)	1983	French	serological	72	113	DR3 B8	NR
Stenszky V	(71)	1986	Hungarian	serological	196	380	A1 DR3 B8	NR
Kendall-Taylor P	(72)	1988	British	serological	127	500	B8 DR3	B17
Semana G	(73)	1990	French	Serological RFLP	287 42	200 42	DR3 B8 Dw24	NR
Mangklabruks A	(74)	1991	American	RFLP	65	65	DR3 DQw2	NR
Schifferdecker E	(75)	1991	German	serological	110	193	B8 Cw7 DR3	NR
Boehm BO	(80)	1992	German	SSOP	304	3724	DRB3*01:01	DRB3*02:02
Yanagawa T	(77)	1993	American	SSOP	94	75	DQA1*05:01	NR
Ratanachaiyavong S	(81)	1994	British	SSOP	51	166	Haplotype: HLA-DR17/DQ2, DPB1*01:01	NR
Badenhoop K	(82)	1995	German	SSOP	271	271	DQA1*0501	DQB1*0602
Barlow AB	(29)	1996	British	SSOP	127	57	DQA1*05:01	NR
Cuddihy RM	(83)	1996	American	SSOP	101	117	independent significance of DQA1*05:01 not confirmed	NR
Kontopoulos A	(84)	1996	Greek	SSOP	105	170	B*39 DRB1*16:01	DRB1*14:01 DQA1*01:04
Lavard L	(85)	1997	Danish	SSOP	90 children	192	DRB1*03:01 DQA1*05:01	DRB1*07:01 DQA1*02:01
Heward JA	(6)	1998	British/Irish	PCR-SSP	228	364	DRB1*03:04 DQB1*02 DQB1*03:01 DQB1*03:04 DQA1*05:01	NR
Chen QY	(86)	1999	American	SSP	92	192	DRB3*02:02 DRB3*01:01 (early onset) DRB3*02:02 (later onset)	DRB1*07
Zamani M	(30)	2000	Belgian	SSOP	101	205	DRB1*03:01 DQA1*05:01	DRB1*07:01 DQA1*02:01
Ban Y	(87)	2002	American	RFLP	60	135	DR3	NR
Simmonds MJ	(88)	2005	British	SSP	871	621	Haplotype DRB1*03-DQB1*02-DQA1*05:01 DRB1*08	Haplotype: DRB1*07- DQB1*02- DQA1*0201
Simmonds MJ	(89)	2007	British	SSP	773	621	B*08 C*07	B*44 C*16 C*03
Bernecker C	(90)	2013	Ferman	Flow cytometry	75	60	none	A2
Martin S	(91)	2014	Romanian	SSOP and SSP	77	445	DRB1*03 DRB1*11	DRB1*01 DRB1*15
Vita R	(5)	2017	Italian	Serological PCR-SSO	58 20/58	130	B8, Cw7, DR3, DR4, DQ2 C*07 C*17 DRB1*04	B14
Zawadzka-Starczewska K	(13)	2022	Polish	NGS	159	2217	B*08:01 B*39:06 B*37:01 C*07:01 C*14:02 C*03:02 C*17:01 DRB1*03:01 DRB1*11:01 DRB1*13:03 DRB1*01:03 DRB1*14:01 DQB1*03:01 DQB1*02:01	B*07:02, C*07:02 C*03:04 DRB1*07:01 DQB1*02:02 DQB1*03:03

NR, not reported; RFLP, restriction fragment length polymorphism; SBT, sequence based typing; SSOP, sequence specific oligonucleotide probe; SSP, sequence specific primers; NGS, next generation sequencing.

Similarly to Asian population, there were discrepancies in the applied methods, group size and analyzed MHC classes (13, 63–91). Most of the studies included only one MCH class, mainly class 2, while some studies included even only one gene, predominantly HLA-DRB1 (**Table 4**). Therefore, due to difficulties in direct comparison of the results, we analyzed numbers of studies which revealed a given allele as a high risk one or a protective one. In order to obtain the most reliable results we included studies which used methods which allowed to obtain allelic specificity, or studies which reported allele group concordant with results in which a specific allele was provided (e.g. DRB1*03 and DRB1*03:01/04). We did not include any of the results obtained by serological methods.

Our present analysis demonstrated that the following alleles were most commonly reported as related to high risk of GD in Caucasians: DQA1*05:01 (7 studies), DRB1*03, including DRB1*03:01/04 (6 studies), C*07/C*07:01 (3 studies), DQB1*02/DQB1*02:01 (3 studies) (**Table 5**). On the other hand, the following alleles were reported as potentially protective: DRB1*07/DRB1*07:01 (5 studies), DQA1*02:01 (3 studies), C*03/C*03:04 (2 studies), DQB1*02/DQB1*02:02 (2 studies) (**Table 5**).

**Table 5:** Alleles reported as Graves’ disease (GD)-related in more than one study in Caucasian population.

GD risk alleles	No of papers	Ref.	GD protective alleles	No of papers	Ref.
DQA1*05:01	7	(6, 29, 30, 77, 82, 85, 88)	DRB1*07/DRB1*07:01	5	(13, 30, 85, 86, 88)
DRB1*03 *DRB1:03:01* DRB1*03:04	2 3 1 (total 6)	(6, 13, 30, 85, 88, 91)	DQA1*02:01	3	(30, 85, 88)
C*07/C*07:01	3	(5, 13, 89)	C*03/C*03:04	2	(13, 89)
DQB1*02/DQB1*02:01	3	(6, 13, 88)	DQB1*02/DQB1*02:02	2	(13, 88)
B*08/B*08:01	2	(13, 89)			
B*39/B*39:06	2	(13, 84)			
C*17/C*17:01	2	(5, 13)			
DRB1*11/DRB1*11:01	2	(13, 91)			
DRB3*01:01	2	(80, 86)			
DQB1*03:01	2	(6, 85)			

The significance of HLA-DRB1*03:01 as GD high risk allele is the only one which is common for both Asian and Caucasian populations. The alleles HLA-DRB1*03:01, DQA1*05:01 and DQB1*02:01 are in strong linkage disequilibrium in Caucasian population (58). Therefore, their significance cannot be considered entirely independent, however, the single presence of any of them can be correlated with the risk of GD. Moreover, some studies demonstrated a common association of HLA-B*08:01, which was also reported as GD high risk allele, with DRB1*03:01 and DQB1*02:01 (6, 13, 29, 30, 77). In our recent study, the combination of these three alleles occurred in 22% of patients with GD, while it was found only in 5.87% of the healthy control group (13). Interestingly, in patients in whom HLA-B*08:01 was present, it occurred with alleles other than the ones described above only in 1.9% of GD patients (13). HLA-B*08:01 is in linkage disequilibrium with HLA-C*07:01 – another high risk allele, whose significance in GD was also demonstrated (92, 93).

Therefore, it can be concluded that HLA-B*08:01, C*07:01, DRB1*03:01, DQA1*05:01, DQB1*02:01 and can be considered the most important predictors of GD development in Caucasians.

On the other hand, HLA-DRB1*07:01 was the allele most commonly demonstrated to be a protective one. Interestingly, this observation is the only one consistent with results of studies in Asians in regard to the protective alleles. It should be indicated that HLA-DRB1*07:01 is in linkage disequilibrium with two other potentially protective alleles – HLA-DQB1*02:02 and DQA1*02:01 (58). *HLA*-C*03:04, which is in no linkage disequilibrium with other discussed alleles, seems to be the only entirely independent protective allele (34, 92). Therefore, the alleles HLA-C*03:04, DRB1*07:01, DQB1*02:02 and DQA1*02:01 can be considered a group of GD protective alleles.

Some differences in the results between Caucasian and Asian populations seem unexpected. Among the alleles, which are in linkage disequilibrium with HLA-DQA1*05:01, only DRB1*03:01 was proved to be GD-related in both Asian and Caucasian populations (13, 30, 35, 54, 91). However, these linkage disequilibrium-based correlations are not exclusive for the Caucasian population, but are common in all analyzed populations, including Asians (58). Therefore, it remains unexplained why these correlations found in Caucasians are absent in Asians, in whom completely different alleles were reported as high risk of GD development. Furthermore, on the basis of the present review, a phenomenon of opposite roles of HLA-DQB1*02:01 can be observed. This allele was demonstrated as high risk in Caucasians (6, 13), but it was also reported as protective in Asian pediatric studies (48, 49). This population-dependent opposite correlation indicates the potential significance of other factors influencing GD risk in either population. Further studies with application of high resolution methods are required to solve this puzzle.

#### 3.3.2 HLA and GO

Similarly to the current state of art in Asians, the results concerning HLA associations with GO development in Caucasians are inconsistent (**Table 6**). From the first study in 1980, DR3 antigen was being demonstrated in serological studies to be not only associated with GD high risk, but also with GO development (66). However, several studies, using different methods, both serological and genetic, did not confirmed such association (36, 72, 97) and the results were highly divergent (80, 94–99). Even a quite recent study performed by Yin et al. did not confirm the existence of HLA-related susceptibility to GO in the group of patients with GD and postulated the importance of environmental or epigenetic factors only (99). However, the authors of that study, similarly to some other earlier ones, focused only on the frequency of *HLA-DR3*, without assessing the frequencies of other alleles, and applied low resolution method. Recently, the significance of HLA-DRB1*03:01 was demonstrated by our research team with application of NGS method (15). We performed genotyping of both MCH classes and found that the frequency of several alleles is significantly higher in GO group as compared to either patients with GD without GO or healthy controls. Except for the previously mentioned HLA-DRB1*03:01, this group of alleles includes HLA-B*08:01, B*39:06, B*37:01, C*07:01, C*14:02, C*03:02, C*17:01, DRB1*11:01, DRB1*13:03, DRB1*01:03, DRB1*14:01, DQB1*03:01, DQB1*02:01. On the other hand, alleles HLA-C*04:01, C*03:04, C*07:02 and DRB1*15:02 were significantly less frequent in GO patients as compared to GD without GO or controls (15). Unfortunately, it was the first study which applied NGS in Caucasian patients, and therefore – similarly to the studies on GD described above – no comparison with other NGS-based studies is possible. The overview of studies focused on associations between HLA and GO in Caucasian is presented in **Table 6**.

**Table 6:** Associations between HLA and Graves’ orbitopathy in Caucasian population.

First author	Ref.	Year	Population	Method	No. of GO patients	No. of GD patients	Nr of healthy controls	High risk HLA	Protective HLA
Farid	(66)	1980	Canadian	serological	24	53	–	DR3	NR
Frecker M	(94)	1986	Hungarian	serological	52	55	–	Genotypes B8-DR3 B8-DR7	DR7 without B8
Schifferdecker E	(75)	1991	German	Serological	84	26	–	No significant correlation	No significant correlation
Boehm BO	(80)	1992	German	SSOP	72	100	223	Heterozygotes DRB3*01:01/*02:02	NR
Badenhoop K	(95)	1996	German	SSOP	135	124	229	significance of DQA1*05:01 not confirmed	NR
Kendall-Taylor P	(72)	1988	British	serological	60	67	500	none	none
Frecker M	(96)	1988	Canadian	serological	64	69	140	B8 DR7 DR3	DR4
Weetman AP	(97)	1988	British	RFLP DR3only	53	51	90	significance of DR3 not confirmed	NR
Villanueva R	(98)	2000	American	SSP	61	40	121	none	none
Yin X	(99)	2012	American	RFLP DR3 only	156	90	–	significance of DR3 not confirmed	NR
Stasiak M	(15)	2023	Polish	NGS	70	91	2217	A*01:01 A*32:01 B*37:01 B*39:01 B*42:01 C*08:02 C*03:02 DRB1*03:01 DRB1*14:01 DQB1*02:01	C*04:01 C*03:04 C*07:02 DRB1*15:02

RFLP, restriction fragment length polymorphism, SSOP, sequence specific oligonucleotide probe; SSP, sequence specific primers; NGS, next generation sequencing; NR, not reported; -, no control group included.

In our study, based on NGS method, HLA-DQB1*02:01 was also found to be a high risk allele (15). Such phenomenon could be expected as this allele is in linkage disequilibrium with HLA-DRB1*03:01 (58). Interestingly, another allele demonstrated to be GO-related in our study – i.e. *HLA*-DRB1*14:01, was previously observed to be GO-related in Japanese patients (60) (**Table ​1**). This similarity is the only one between our results in Caucasian population and the already published data for Asian population in regard to GO. Nevertheless, such a lack of coherence between Asians and Caucasians could be expected, because HLA-related susceptibility for many autoimmune diseases differs between these two populations. In our NGS-based study, the highest risk of GO was associated with the presence of HLA-C*08:02 (OR 6.9) and -B*37:01 (OR 4.5) as well as DRB1*14:01 (OR 6.2) (15). There is no linkage disequilibrium between these three alleles (58, 92, 93), so the presence of any of them constitutes independent high risk factor.

Interestingly, in the same study, we demonstrated a protective effect of HLA-C*04:01 allele, which was previously reported as related to increased risk of subacute thyroiditis (SAT) (100, 101). This coincidence can be considered an explanation of the fact that SAT and GO actually occur together extremally rarely in clinical practice. It is worth noting that HLA alleles had already been suggested to be related with the course of SAT and GD in patients with simultaneous presence of both these diseases (102).

### 3.4 Other associations

#### 3.4.1 Asian population

##### 3.4.1.1 HLA and GD course, comorbidities and relapse

In a large study performed in Taiwanese patients with GD, significant associations between HLA and comorbidities were found (40). In this group, genotypes HLA-A*11:01-A*33:03 and A*02:07-A*11:01, as well as HLA-B*40:01-B*58:01 were significantly correlated with heart disease. Additionally, genotypes HLA-A*24:02-A*24:02 and HLA-B*46:01-B*46:01 were significantly associated with stroke, while genotypes HLA-B*46:01-*46:01 and DPA1*01:03-DPA1*01:03 as well as DPB1*02:01-DPB1*05:01 were correlated with hypertension. On the other hand, genotypes HLA-DPA1*01:03-DPA1*01:03 and DPA1*01:03-DPA1*02:01 were significantly more frequent among the subjects with GD and diabetes than in those with GD without diabetes (40).

Some studies suggested that the age of GD onset is HLA-dependent. As it was indicated above, it was postulated that HLA-Bw46 is associated with younger age of GD onset (32, 34), while B5 was suggested as related to older age of onset (32). Few years later, Onuma et al. published entirely opposite results and stated that HLA-B46 is related to late onset of GD, while DPB1*05:01 is associated with early onset (47). In Japanese and Chinese studies, which included children only, DQB1*03:03 was demonstrated to be GD-related (48, 49). However, in a recent study performed in Korean children, previously postulated significance of HLA-B*46:01 was confirmed, and – additionally – HLA-C*01:02, DPB1*02:02, DPB1*05:01, occurred to be significantly associated with GD (14).

In a recent study by Azizi et al. with application of SSP method, DQB1*05 HLA polymorphism was demonstrated to be related to GD relapse (103). The authors analyzed relapse rates in patient during 48 months period after methimazole withdrawal (103). Unfortunately, no other similar study in Asians is available for comparison.

#### 3.4.2 Caucasian population

##### 3.4.2.1 HLA and GD recurrence and course

In Caucasian population, HLA-DQA1*05 variant was demonstrated to have ability to predict relapse. Surprisingly, combinations with other HLA risk genes forming the risk haplotype DRB1*03-DQA1*05-DQB1*02 did not improve the predictive value (104). Several years earlier, no significance of HLA DQA1*05:01 in GD recurrence was reported by Badenhoop et al. (95) However, a study in which patients were observed for two years after antithyroid drug withdrawal revealed that HLA-DRB1*03, DQA1*05, and DQB1*02 polymorphisms are strong predictors for recurrence after antithyroid drug therapy (105). There is a strong linkage disequilibrium between the them and their significance in GD relapse in Caucasians should be considered.

Most of the studies focused on GD recurrence used serological methods. In a study performed by Shifferdecker et al. in German population, HLA DR5 was associated with relapse of GD, whereas HLA DR7 and B12 were negatively correlated with relapse (75). A role of B8 was postulated by Irvine et al, who observed significantly more common relapses in B8 positive patients (106). Another study demonstrated significance of HLA-DR3 haplotype and lack of association with HLA-B8 haplotype and GD relapse (107). Some studies underlined the role of HLA-DR3 (65, 108), while other did not confirmed this association (109). Similarly, de Bruin et al. found no significance of HLA-DR3, but demonstrated that HLA-Cw7 was associated with relapse, while HLA-DR4 was protective against recurrence (110). In a study by Young et al., neither HLA-B8 nor HLA-DR3 conferred increased likelihood of relapse (111). Significant association of GD relapse and DQA2 genotype was found with application of RFLP method (112). Most of the authors did not find any correlation between HLA and GD recurrence (113–117).

Few studies analyzed the severity of GD in regard to HLA. Preus et al. demonstrated that patients with severe course were characterized by a high frequency of HLA-A1 andHLA-B8, while mild course patients showed a higher frequency of HLA-B12 (118). Some studies focused directly on the HLA associations with GD onset. In a study by Lavard et al. DRB1*03 and DRB3*01:01 were associated with juvenile GD onset (<20 years of age), whereas DRB3*02:02 was associated with adult onset of the disease (85). Other clinical course-related associations demonstrated that HLA-DR3 positive GD patients were also found to be more resistant to radioiodine therapy than patients negative for these antigens (66). Additionally, patients with GD and haplotype HLA-DRB1*03/DRB1*11 were found to have higher FT4/TT3 ratio and anti-thyroglobulin antibody levels (91).

Unfortunately, most of studies which analyzed GD recurrence or the disease course were performed decades ago and applied serological methods. Therefore, the described discrepancies in the obtained results were not unexpected, and currently, no clear correlation in regard to HLA and GD severity or age of the disease onset can be confirmed. Further studies using high resolution methods may provide conclusive results.

##### 3.4.2.2 HLA and non-genetic risk factors of GO

Increased level of total cholesterol (TC) and/or – of low-density lipoprotein cholesterol (LDL) is generally known to increase the risk of GO. Very recently, our research team demonstrated a significant correlation between the higher TC/LDL levels (11) and the occurrence of GO-related high-risk alleles (HLA-B*37:01 and C*03:02). Moreover, the presence of alleles associated with GD without GO (HLA-C*17:01 and B*08:01), as well as alleles which are in linkage disequilibrium with B*08:01 (i.e., HLA-DRB1*03:01 and DQB1*02:01), was shown to be correlated with lower TC levels (11). These results seem important as they provide evidence that correlations between TC/LDL and GO can be HLA-dependent and confirm the relevance of TC/LDL lowering therapy in the cases where the risk of the development of GO is significant.

#### 3.4.3 Significance of HLA amino acid variants in Asians and Caucasians

The significance of amino acid variants of HLA molecules were also postulated in the context of risk of GD development. In a study by Shin et al., both Leu35 (OR = 23.38, P = 0.0002) and Glu55 (OR = 23.38, P = 0.0002) of HLA-DPB1 were strongly associated with GD (14). These authors concluded that amino-acid signatures of the HLA-DP β chain, might contribute to the molecular pathogenesis of GD (14). However, in Japanese population, amino acid variants in HLA-DRB1 allotypes were examined, and critical significance of position 9 amino acid variants for GD development was found (57). Glu-9 variant, which was reported as related to GD high risk, is encoded by HLA-DRB1*03:01, DRB1*04:05 and -DRB1*14:03 alleles, which confer susceptibility to GD in several studies. On the other hand, Cys-9 variant, which was reported as protective against GD, is encoded by HLA-DRB1*07:01, DRB1*01:01 and DRB1*15:02 alleles, which were demonstrated to be protective against GD (57). It is speculated that changes in the charge and polarity of amino acids at these positions modify the three-dimensional structure of the DR peptide-binding pocket, which leads to susceptibility to or protection against GD (57, 119). Negatively charged Glu-9 was demonstrated to confer susceptibility to GD, while uncharged Cys-9 conferred protection against GD (57).

It was postulated that HLA-DR3 (HLA-DRB1*03) Arg-74 is the critical amino acid for the development of GD (88, 120). The group HLA-DR3 includes more than 30 alleles. A HLA-DRβ (beta chain of HLA-DR3) pocket variant with arginine at position 74 (HLA-DRβ-Arg74) is associated with an increased risk of GD, while glutamine (HLA-DRβ-Gln74) variant was protective (88, 119, 120). Three-dimensional computer modeling of the HLA-DR pocket allowed proving that electrostatic potential of HLA-DRβ-Arg74 generates a more positively charged P4 HLA-DR pocket than HLA-DRβ-Gln74. Therefore, the susceptibility to GD may be related to electrostatic potential of HLA-DR pocket and positive charge can be a high risk factor. The significance of this phenomenon can be confirmed by the finding that, among DRB1 molecules, DRB1*03 contains a positively charged Arg at β74, and DRB1*07 contains a non-charged Gln at β74 position (88). This positive charge difference is believed to facilitate auto-antigen presentation and T-cell activation because of its increased binding affinity of pathogenic self-peptides to the HLA-DR pocket (121). Candidate pathogenic peptides in GD include peptides derived from TSHR and Tg. This interaction between peptides of thyroidal origin and the HLA-DRβ-Arg74 pocket was considered as potential therapeutic target (121). Such an antigen-specific immunotherapy may constitute a crucial progress in the GD therapy, as current treatment modalities do not reverse the autoimmune process. It is well known that TSHR is the major autoantigen in GD. A TSHR peptide, designated TSHR.132, was found to be a dominant TSHR peptide in GD (122–124). TSHR.132 was demonstrated to bind with high affinity to recombinant HLA-DRβ-Arg74 and to cells that express HLA-DRβ-Arg74 (121). Cepharanthine is a small molecule compound which was demonstrated to inhibit T-cell activation by TSHR.132 ex vivo in splenocytes isolated from humanized mice induced with EAGD (experimental autoimmune Graves’ disease) (123, 124). Cepharantine directly interacts with Arg74 and blocks the HLA-DR3 pocket variant associated with AITD including GD. Due to the significant role of HLA-DR3 in many autoimmune diseases, cepharantine may be a potentially efficient causative therapy for GD and other autoimmune disorders such as Addison’s disease, Hashimoto’s thyroiditis, myasthenia gravis or SLE (121, 123, 124).

Additional various HLA polymorphisms were also described as associated with GD. In two independent Caucasian populations, an intronic variant (rs3094228) in HLA complex P5 (HCP5) was demonstrated to be associated with GD susceptibility and age of onset, which indicates a potential role of long non-coding ribonucleic acids, including HCP5, in GD pathogenesis (125, 126). HCP5 gene polymorphism (rs3094228) was associated with an earlier age of GD onset and patients with higher number of the HCP5 risk alleles tend to have a significantly earlier onset of GD (126).

## 4 Conclusions

In Asian population, GD was found to be associated mostly with HLA-B*46:01, DPB1*05:01, DRB1*08:02/03, DRB1*16:02, DRB1*14:03, DRB1*04:05, DQB1*03:03 and DQB1*05:02, while DRB1*07:01, DRB1*01:01, DRB1*13:02, DRB1*12:02 are potentially protective. HLA-B*38:02, DRB1*16:02, DQA1*01:02, DQB1*05:02 can be considered associated with increased risk of GO in Asians, while HLA-B*54:01 may play protective role. In Caucasians, C*07:01, DQA1*05:01, DRB1*03, DQB1*02:01, are associated with GD risk while DRB1*07:01, DQA1*02:01 may be protective. Data are scarce in regard to GO in Caucasians, but HLA-B*08:01, B*39:06, B*37:01, C*07:01, C*14:02, C*03:02, C*17:01, DRB1*03:01, DRB1*11:01, DRB1*13:03, DRB1*01:03, DRB1*14:01, DQB1*03:01, DQB1*02:01 were found more frequently while alleles HLA-C*04:01, C*03:04, C*07:02 and DRB1*15:02 were significantly less frequent in GO patients as compared to GD without GO, or to controls. HLA polymorphisms potentially influence the course of GD, its recurrence risk, comorbidities and a presence of GO-related non-genetic risk factors. Further studies based on NGS methods are required to clearly demonstrate or confirm the correlations, as identification of an actual set of high risk and protective alleles in a given population can constitute a reliable tool for the individual risk assessment. Additionally, it may contribute to potential development of HLA-based treatment modalities.

## Data availability statement

The original contributions presented in the study are included in the article/supplementary material. Further inquiries can be directed to the corresponding author.

## Author contributions

MS: Conceptualization, Data curation, Formal Analysis, Funding acquisition, Investigation, Methodology, Project administration, Resources, Supervision, Visualization, Writing – original draft, Writing – review & editing. BS: Formal Analysis, Investigation, Methodology, Resources, Software, Writing – original draft. KZ-S: Investigation, Resources, Writing – review & editing. AL: Methodology, Project administration, Resources, Validation, Writing – review & editing.
